# Increased Urinary Exosomal MicroRNAs in Patients with Systemic Lupus Erythematosus

**DOI:** 10.1371/journal.pone.0138618

**Published:** 2015-09-21

**Authors:** Javier Perez-Hernandez, Maria J. Forner, Carolina Pinto, Felipe J. Chaves, Raquel Cortes, Josep Redon

**Affiliations:** 1 Genotyping and Genetic Diagnosis Unit, INCLIVA Biomedical Research Institute, Valencia, Spain; 2 Cardiometabolic and Renal Unit, INCLIVA Biomedical Research Institute, Valencia, Spain; 3 CIBER of Diabetes and Associated Metabolic Diseases (CIBERDEM), Institute of Health Carlos III, Minister of Health, Barcelona, Spain; 4 CIBER Physiopathology of Obesity and Nutrition (CIBEROBN), Institute of Health Carlos III, Minister of Health, Madrid, Spain; Mayo Clinic Arizona, UNITED STATES

## Abstract

There is increased interest in using microRNAs (miRNAs) as biomarkers in different diseases. Present in body fluids, it is controversial whether or not they are mainly enclosed in exosomes, thus we studied if urinary miRNAs are concentrated inside exosomes and if the presence of systemic lupus erythematosus with or without lupus nephritis modifies their distribution pattern. We quantified specific miRNAs in urine of patients with systemic lupus erythematosus (n = 38) and healthy controls (n = 12) by quantitative reverse-transcription PCR in cell-free urine, exosome-depleted supernatant and exosome pellet obtained by ultracentrifugation. In control group, miR-335* and miR-302d were consistently higher in exosomes than in exosome-depleted supernatant, and miR-200c and miR-146a were higher in cell-free fraction. In lupus patients, all urinary miRNAs tested were mainly in exosomes with lower levels outside them (p<0.05 and p<0.01, respectively). This pattern is especially relevant in patients with active lupus nephritis compared to the control group or to the SLE patients in absence of lupus nephritis, with miR-146a being the most augmented (100-fold change, p<0.001). Among the exosomal miRNAs tested, only the miR-146a discriminates the presence of active lupus nephritis. In conclusion, urinary miRNAs are contained primarily in exosomes in systemic lupus erythematosus, and the main increment was found in the presence of active lupus nephritis. These findings underscore the attractiveness of exosomal miRNAs in urine, a non-invasive method, as potential renal disease markers.

## Introduction

Over the last few years, there has been increasing interest in detecting body fluid micro RNAs (miRNAs) as biomarkers of activity in several diseases. MiRNAs, a family of small non-coding RNAs, play an important role in a variety of biological processes [1–3], mainly through their regulation of post-transcriptional gene expression. Therefore, changes in the profile of cellular miRNAs have been shown to correlate with different pathophysiological conditions and could be specific to particular disease states [2,4–6]. Tissue-specific miRNAs are secreted into blood and other biological fluids, such as urine [7].

Extracellular miRNAs released into urine can circulate bound to RNA-binding proteins or packaged into microvesicles such as exosomes [8–11]. Exosomes are small membrane vesicles with a size of 30–100 nm that are secreted by different cell types [12]. They can be isolated from a variety of body fluids, including plasma, urine, saliva, amniotic fluid and breast milk [13,14]. Moreover, it has been recently reported that exosomes were enriched in miRNA, mRNA, small nuclear RNA, transfer RNA and long intergenic RNA [15,16]. This finding sparked the idea that exosomes may represent a new type of intercellular messenger, playing an important role in cell-to-cell communication, and serve as potential biomarkers for diagnosis, prognosis, or predictive response to therapies.

In the kidney, miRNAs are indispensable to the regulatory mechanisms for renal development, maintenance of renal function and homeostasis processes. Likewise, their role in the progression of kidney disease has recently been emphasized [17,18]. Urinary miRNAs may be filtered from the circulation, but are more frequently released from nephron cells actively secreted into exosomes enriched in kidney-specific miRNAs [19,20]. Changes in urinary miRNAs have been reported in several renal diseases, such as nephritic syndrome [8], IgA nephropathy [21], acute or chronic renal injury [22,23], diabetic nephropathy [24] and lupus nephritis (LN) [25]. These results suggest that urinary miRNAs have a strong potential to be biomarkers of renal injury. Recently, Cheng et al characterized the miRNA content of exosomes and non-exosomal fractions isolated from urine in healthy volunteers by deep sequencing [26], and Lv et al., showed that high levels of miRNA were confined to the urinary exosomes in patients with a diversity of chronic diseases [9]. These results provided the basis for identifying miRNA biomarkers in human urine. There is no evidence, however, supporting the relative contribution of exosomal miRNAs to whole miRNAs in urine and the effect of renal disease on the miRNAs distribution in the different urine fractions nor their clinical meaning. In the present study, the miRNA pattern in different stages of SLE are investigated using quantitative reverse-transcription PCR (RT-qPCR) in cell-free urine (CFU), exosome-depleted supernatant (Sn) and exosome pellet (Exo).

## Material and Methods

### Ethics Statement

The study protocol was approved by the Ethics committee of the Hospital Clinico Universitario of Valencia in accordance with the Declaration of Helsinki of 1975 as revised in 2008. All subjects have signed a written informed consent.

### Patient and Urine Sample Processing

The study included 28 consecutive patients with systemic lupus erythematosus (SLE) (6 active LN, 10 inactive LN and 12 absence of LN). SLE and Lupus nephritis were diagnosed following the KDIGO Clinical Practice Guideline for Glomerulonephritis diagnostic criteria [27], the presence of LN were considered with impaired kidney function with proteinuria (>0.5 g/day or >1 g/day with previous proteinuria) or an active urine sediment, that includes hematuria, especially leukocyturia in the absence of infection, and red and white blood cell casts, or kidney biopsy with active glomerulonephritis. Inactive LN was considered when the patients decreased the proteinuria levels (< 0.5 g/day) and had inactive urinary sediment with stable kidney function. Clinical and laboratory data including, serum creatinine, serum complement levels, 24h-proteinuria, and Systemic Lupus Erythematosus Disease Activity Index (SLEDAI) were recorded. Glomerular filtration rate was estimated by the MDRD equation [28]. We also studied the urine from 12 healthy volunteers as controls which had normal renal function, normal urinalysis, and no history of urinary tract infection, renal stone, and other renal or genitourinary disease. Fresh first morning urine samples (100 mL) were collected in sterile containers and were processed within 1 h after collection.

### Isolation of the different urinary fractions by sequential differential centrifugation

Urinary cells and debris were removed by centrifugation at 2250 g for 30 min at 4°C, obtaining the first fraction, cell-free urine. Then, 100 mL of the collected supernatant were transferred to clean tubes with 4.2 mL of protease inhibitor cocktail (Sigma; USA) and was centrifuged at 20,000 g_av_ for 45 min at 4°C to eliminate large microvesicles (Ultracentrifuge Optima L 100K, 70 Ti rotor, Beckman Instruments, USA). The supernatant was spun in an ultracentrifuge at 160,000 g_av_ for 70 min at 4°C, obtaining exosome-depleted supernatant. Exosome pellets were washed with 2 mL of sterile RNase-free PBS and ultracentrifuged again at 160,000 g_av_ for 70 min (Ultracentrifuge Optima L 100K, 70.1 Ti rotor, Beckman Instruments, USA). A 50 mL-pellet was resuspended in 100 μL of sterile PBS for protein quantification and electron microscopy and the other 50 mL-pellet was resuspended in 100 μL of Exosome Resuspension Buffer (Invitrogen, Life Technologies, USA) for RNA isolation. The three urine fractions: cell-free urine (CFU), pellet containing exosomes (Exo) and the exosome-depleted supernatant (Sn) were then immediately processed to extract RNA.

### Isolation of RNA

RNA was extracted from cell-free urine (CFU) and exosome-depleted supernatant (Sn) fractions using the mirVana PARIS kit (Ambion, Life Technologies, USA) using the manufacturer’s recommended protocol for total RNA. Briefly, 500 μL of sample were mixed with an equal volume of 2x Denaturing Solution and 1 mL of Acid phenol-chlorophorm. After centrifugation at 10,000 x g for 5 min, the aqueous phase was transferred to a new tube and, using silica-membrane RNeasy spin columns, purified RNA was extracted in 100 μL of elution buffer and stored at -80°C. From exosome pellets, RNA was extracted using the Total exosome RNA and protein isolation kit (Invitrogen, Life Technologies, USA) from 100 μL of exosome suspension and stored at -80°C. To normalize the technical variability (sample-to-sample variation), we spiked-in a synthetic and non-homologous to human C. elegans miRNA, cel-miR-39 (Qiagen; TaqMan ID: 000200), after the addition of the denaturing solution to the samples. This miRNA, went through the entire RNA isolation and was measured by RT-qPCR, providing an internal control. To confirm that the RNA was confined to the exosomes, urinary exosomes were treated with 0.1 μg μl-1RNase A (Biosharp). Quantification of total RNA was performed by NanoDrop ND-1000 (Thermo Fisher Scientific, USA), whereas quality and size distribution were examined by capillary electrophoresis (Agilent 2100 Bioanalyzer, Agilent Technologies, Santa Clara, CA, USA) with the RNA 6000 Pico chip.

### Reverse transcription and microRNA quantification

Because a consensus internal reference is lacking for the RT-qPCR analysis of serum or urine miRNAs, concentrations were normalized to the sample volume as described [29]. Five μL of total RNA eluate was used as a template in a 15 μL reverse transcription (RT) reaction using the TaqMan microRNA assay and Reverse Transcription kit (Applied Biosystems, USA). A fixed volume of cDNA, 1.33 μL, was combined with TaqMan universal PCR master mix II no UNG and specific TM microRNA assay primers. All reactions were run in triplicate, including blank/negative controls without cDNA, and were performed by using the LightCycler 480 II real-time PCR system (Roche). Data was analysed with the LightCycler 480 software (v1.5), determining the threshold cycle (Ct) and the average of each sample. The following TaqMan microRNA assays were used to measure four specific target microRNAs: miR-335* (ID 002185) and miR-302d (ID 000535), miR-200c (ID 002300), and miR-146a (ID 000468). Candidate miRNAs were selected from literature search based on their presence in urine, also miR-200c were upregulated in kidney disease and miR-146a glomerular expression was increased in lupus nephritis [7,30,31]. We have analysed if SLE with or without renal disease affects these selected miRNAs levels in the same way.

The target microRNA data were normalized across samples using a median normalization procedure [29]. For each sample, the average Ct of cel-miR-39 was calculated. Then, the median of all the mean cel-miR-39 Cts considering all samples was designated as Median_Spike In_Ct value. To be compared, the final Ct for a target microRNA in each sample was adjusted based on the following formula: Normalized_Ct value for the miRNA in the sample = Raw_average_Ct value—[(Spike In_Average_Ct value of the given sample)—(Median_Spike In_Ct value)]. A Ct of 35 was considered the lower level for detection. In order to calculate the target miRNA differences between fractions of urine and groups, the relative quantity was calculated by the comparative Ct method 2^(-ΔΔCt)^ after normalization to the cel-miR-39.

### Western Immunoblotting

The CFU, Exo and Sn of urine were lysed in RIPA lysis buffer on ice for 20 min, sonicated 5 min and then centrifuged at 16,100 g for 10 min. The supernatant was collected and transferred to a new tube. Total protein quantity were calculated by the Lowry method. An equal amount of total soluble protein (20 μg) were electrophoresed on 4–12% Bis-Tris NUPAGE SDS-PAGE (Life Technologies, USA) and transferred to a PVDF membrane for each sample analysed. The blotting membrane was incubated with exosomal primary antibodies CD9 mouse monoclonal dilution 1:300 (Santa Cruz Biotechnology, USA) and TSG101 mouse monoclonal dilution 1:500 (Abcam, UK), and with non-exosomal primary antibodies: calnexin rabbit polyclonal dilution 1/600 (Abcam, UK), GM-130 rabbit monoclonal dilution 1/1200 (Abcam, UK) and nucleoporin 62 rabbit polyclonal 1/500 (Abcam, UK), followed by incubation with anti-mouse IgG Alkaline Phosphatase peroxidase secondary antibody (Sigma, St Louis, MO, USA). The immunoreactive bands were visualized using an acid phosphatase-conjugated secondary antibody and nitro blue tetrazolium/5-bromo-4-chloro-3-indolyl phosphate (NBT/BCIP, Sigma, USA) substrate system and quantified using the TotalLab TL-100 (v.2008) program.

### Transmission Electron Microscopy (TEM)

We placed a drop, approximately 30 μL of exosomes resuspended in PBS on the paraphilm. Then, a formvar carbon-coated nickel grid was positioned on top of each drop for 15 minutes. Finally, a drop of 2% uranyl acetate was placed on the paraphilm and incubated the grid on top of the drop for 5 minutes in the dark; thus, the grids were negative stained. After air drying, the grids were examined with a CM-10 Phillips transmission electron microscope. The size distribution of captured exosomes in a total of 10 micrographs from two samples was analysed using an image processing program (ImageJ, National Institutes of Health).

### Statistical analysis

The statistical analyses were performed with Statistical Package for the Social Sciences (v 12.1.3 for Windows; IBM SPSS, Chicago, IL, USA). Student’s t-test and Fisher’s exact test were used to determine the difference of clinical characteristics between two groups where appropriate. The miRNA data was presented as the mean (SEM) or as the fold change relative to control group (SEM) and other variables were expressed as the mean (SD). The nonparametric Mann–Whitney *U*-test was used to compare differences in relative fold changes of miRNAs between urinary fractions and disease groups. For exosomal miRNAs, we constructed ROC curves and calculated the area under the ROC curve (AUC) to identify their associations with SLE and lupus nephritis. Frequency tables and ROC curves were then used to evaluate the diagnostic effects and to find the appropriate cut-off point. A *P* value <0.05 was considered statistically significant.

## Results

### Clinical characteristics of the study population

The total SLE patients was slightly older than controls (43 ± 8 compared to 35 ± 6 years, p<0.05), and two groups had similar gender distribution (89% and 77% female, respectively). Table 1 summarizes the clinical features of the study population according to renal dysfunction. The age of participants in the control group was lower than in the lupus nephritis group, active or inactive (p<0.01 and p<0.05, respectively), while the differences among the patient groups were not significant. Furthermore, active LN group had worse renal function with higher proteinuria and GFR than controls (p<0.01), inactive LN and in absence of LN (p<0.05). Baseline serum C3 and C4 levels were also significantly lower in active LN patients.

**Table 1:** Baseline clinical data of the study subjects.

Characteristic	Active LN (n = 6)	Inactive LN (n = 10)	Absence LN (n = 12)	Healthy Controls (n = 12)
**Age**	50.3± 7.7**	42.2 ± 6.6*	40.6 ± 6.9^†^	35.4± 6.5
**Gender (Male/Female)**	1/4	2/8	1/11	2/10
**Serum creatinine (mg/dL)**	0.82 ± 0.11	0.92 ± 0.42	0.77 ± 0.24	0.73 ± 0.13
**GFR (mL/min/1.73 m** ^**2**^ **)**	80.3± 11.5**	83.8± 29.5*	97.7 ± 37.5	106.2± 14.5
**Proteinuria (g/day)**	1.82 ± 0.58**	0.44 ± 0.21^†^	0.12 ± 0.08^†^	0
**C3 (mg/dL)**	40.8± 10.2	72.1 ± 36.9^†^	79.7 ± 25.4^‡^	-
**C4 (mg/dL)**	5.8± 4.1	15.2 ± 11.2^†^	16.6± 9.6^†^	-
**SLEDAI**	9.2 ± 6.6	8.8 ± 7.4	3.8 ± 3.2^†^	-
**Anti-double-stranded DNA (U/mL)**	182.38 ± 137.68	175.91 ± 122.06	112.51 ± 117.23	-

The data are expressed as the mean ± SD, unless noted otherwise. C3: complement 3, C4: complement 4; GFR: glomerular filtration rate; LN: lupus nephritis; SLE: systemic lupus erythematosus; SLEDAI: SLE activity index.

*p<0.05 compared to controls

**p<0.01 compared to controls

†p<0.05 compared to Active Lupus Nephritis

^‡^p<0.01 compared to Active Lupus Nephritis

### Isolation and characterization of exosomes from urine

**Fig 1:**
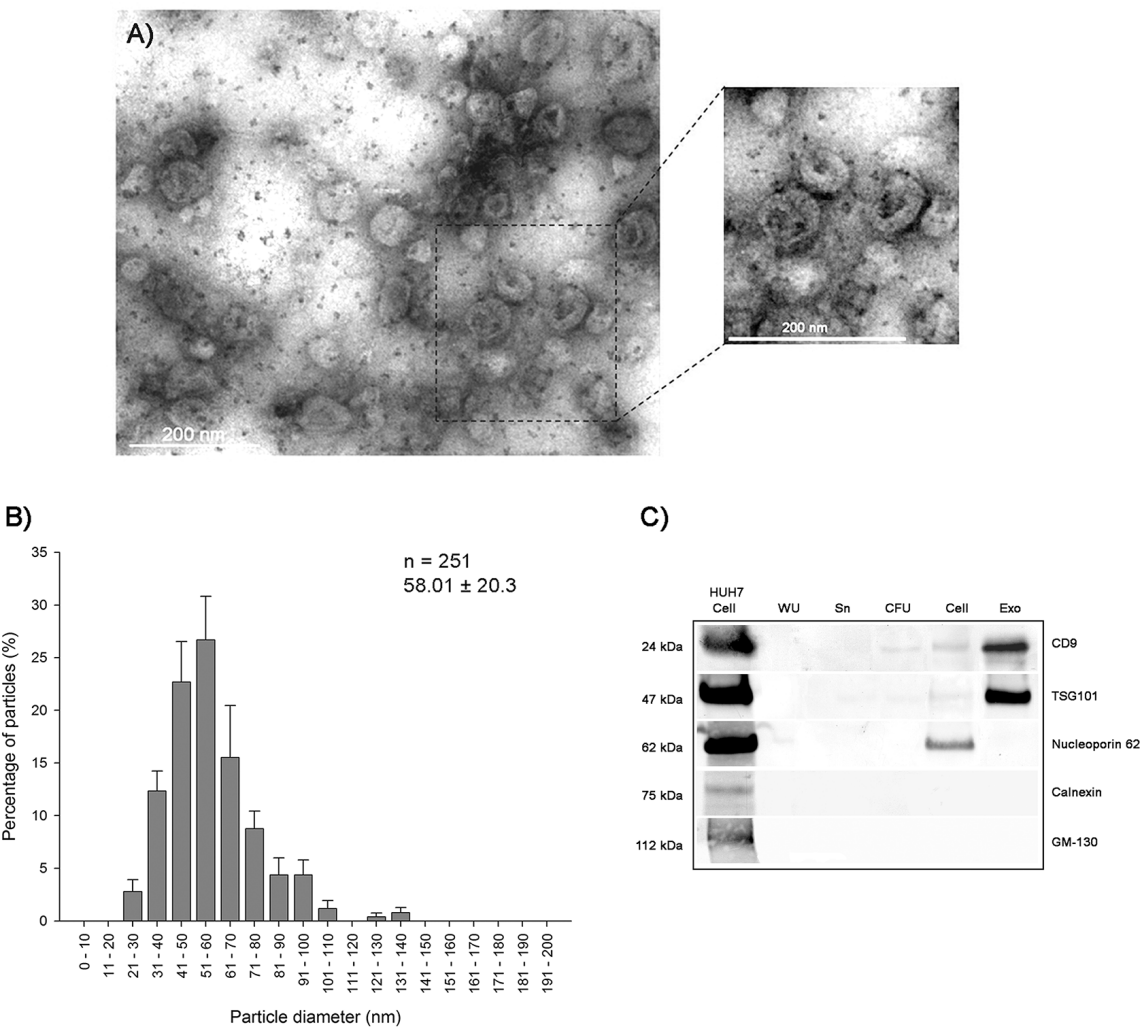
Characterization of urinary exosomes isolated by ultracentrifugation. (A) Transmission electron microscopy (TEM) micrographs of exosome isolations stained by uranyl acetate. Bar represents 200 μm. (B), size distribution (mean ± SD) of urinary exosomes. A total of 10 micrographs for two samples were analysed. (C), Western immunoblotting of exosomes isolated from urine using exosomal markers such as Tsg101 and CD9, and non-exosomal markers such as calnexin (endoplasmic reticulum), nucleoporin p62 (nucleus) and GM-130 (Golgi apparatus) in the individual urine fractions: whole urine (WU), exosome-depleted supernatant from the 160,000 g spin (Sn), cell-free urine (CFU) and exosome pellet (Exo). Whole-cell lysates from HuH7 cells were loaded as a positive control sample.

**Fig 2:**
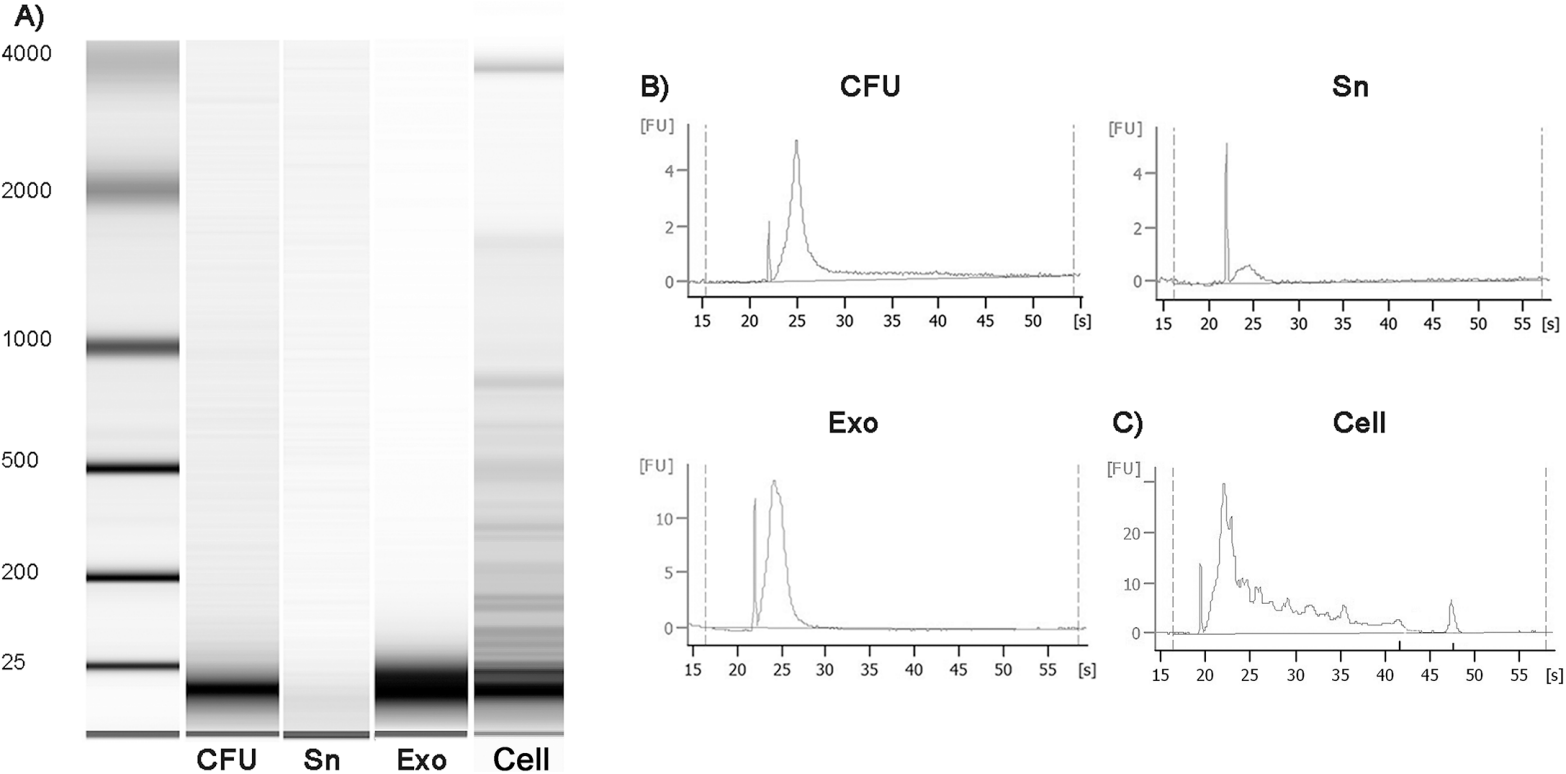
Representative Bioanalyzer profiles of urine fractions using the Agilent RNA Pico Chip. (A), the small RNAs were dominant in the exosomal RNAs. (B), Bioanalyzer electropherograms from the various urine components, cell-free urine (CFU), exosome-depleted supernatant (Sn) and exosomes (Exo). (C), Total RNA cell pellets from urine, as determined also by an RNA Pico Chip. Large RNA species (18S and 28S) were slightly detected in cell pellets, suggesting degradation of cellular RNA.

In order to validate the exosome purification protocol, urinary exosome morphology, shape and size were analysed by transmission electron microscopy. The isolated microvesicles had a spherical shape with a size ranging from 21–140 nm (Fig 1A and 1B). Quantitative analysis revealed that 94.8% of exosomes were between 30–100 nm. Ultracentrifugation Exo, CFU and Sn were also analysed by Western blotting showed that urinary exosome-associated proteins were mainly visualized in the Exo fraction (Fig 1C), confirming that TSG101 and CD9 positive exosomes were recovered with ultracentrifugation. Moreover, we have observed that non-exosomal markers were highly enriched only in HuH7 cellular pellet, with absence in the exosome pellets and the different fractions obtained during the isolation procedure (whole urine, cell-free urine and exosome-depleted supernatant) (Fig 1C). Furthermore, we compared the total protein quantity in the exosome pellets among samples and did not find significant differences, thus the exosome concentration was similar in all samples. In addition, upon analysing the small RNA species (<200 nt) extracted from individual fractions of urine using Agilent Bioanalyzer Pico RNA Kit (Fig 2), the small RNA was found enriched in the exosomal pellet (361 pg/μL) compared to CFU (202 pg/μL) and Sn (41 pg/μL) (Fig 2C). The cellular pellet obtained from whole urine showed a small amount of RNA, the majority of which was degraded cellular RNA (Fig 2C).

### Increased miRNAs in the urinary exosome fraction than the other components of urine

**Fig 3:**
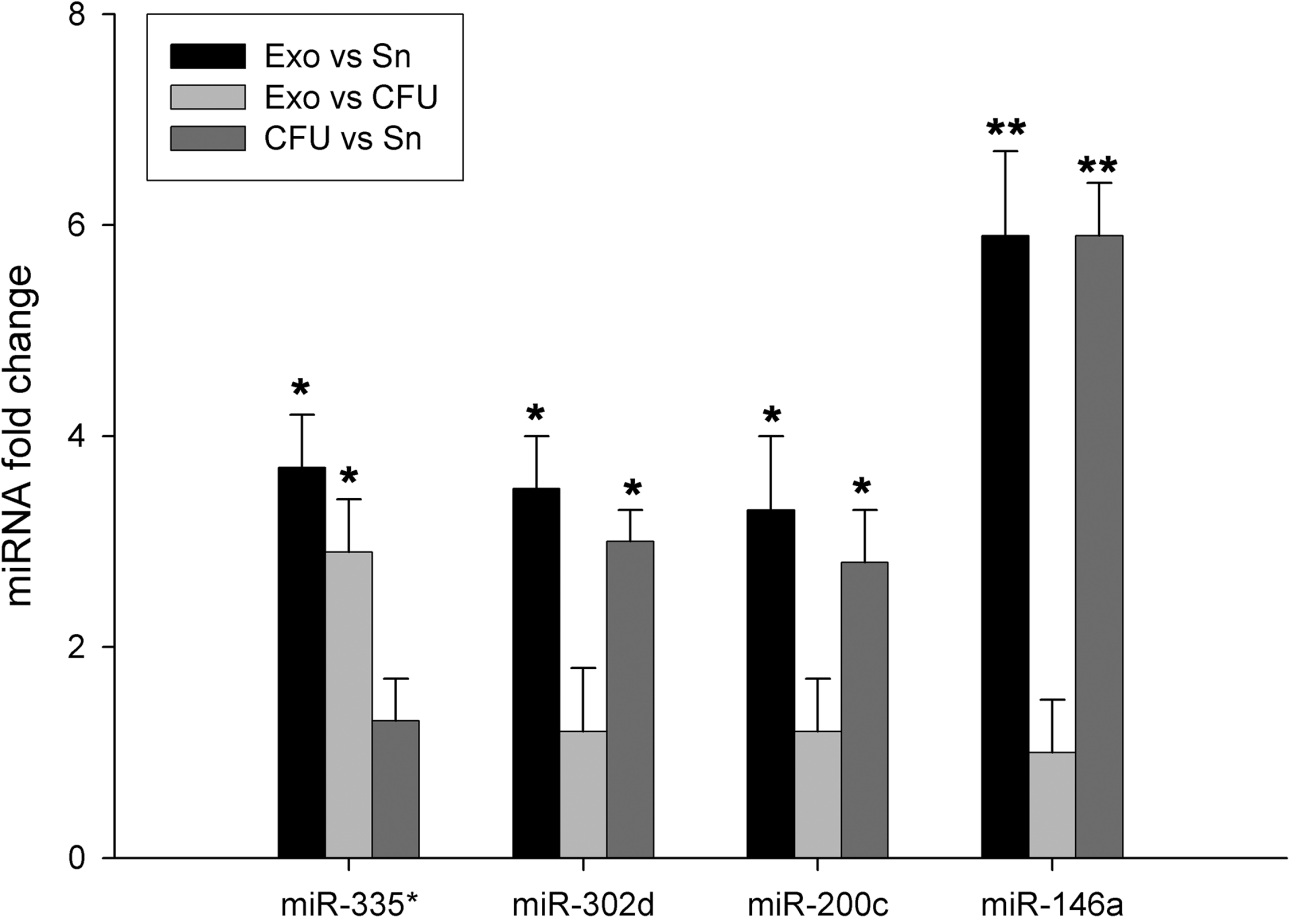
Comparison of fold-change expression of microRNAs between urinary fractions from patients with systemic lupus erythematosus. The level of the four miRNAs species miR-335*, miR-302d, miR-200c and miR-146a was quantitatively assayed and compared between urine fractions, cell-free urine (CFU), exosome-depleted supernatant (Sn) and exosomes (Exo), in systemic lupus erythematosus with individual TaqMan miRNA Assays. Spiked-in cel-miR-39 was used as the normalization control for all samples. The graphs represents relative miRNA fold change by the 2^-ΔΔCt^ method. Data represents the mean ± SEM. *p<0.05; **p<0.01.

**Fig 4:**
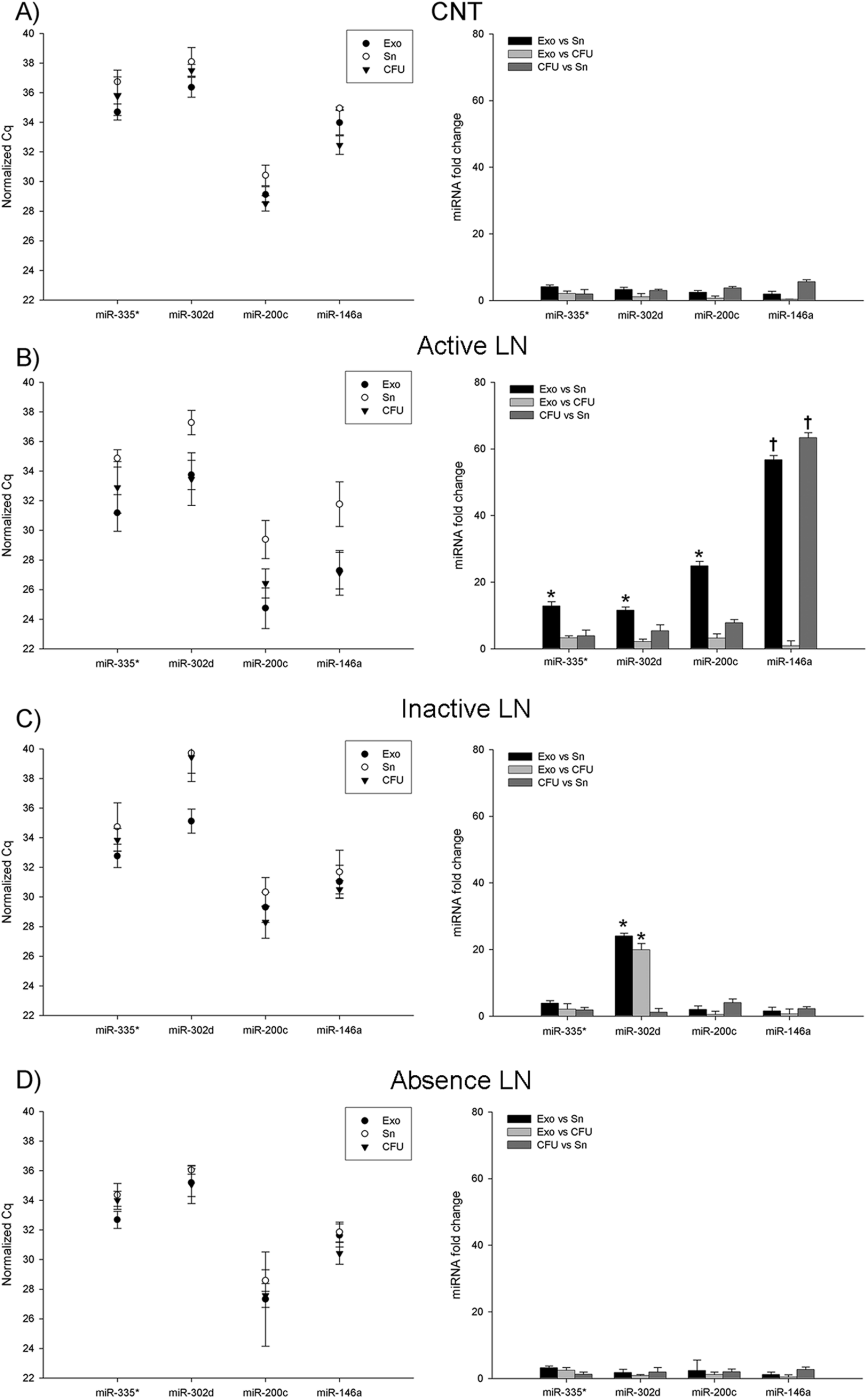
Urinary miRNA quantification and comparison among urine components in systemic lupus erythematosus groups. The level of the miRNAs was compared in the different urine fractions, cell-free urine (CFU), exosome-depleted supernatant (Sn) and exosomes (Exo), in controls (A), active lupus nephritis (B), inactive lupus nephritis (C) and systemic lupus erythematosus with the absence of lupus nephritis (D). Spiked-in cel-miR-39 was used as the normalization control for all samples. Left data graphs are expressed as absolute differences (Ct) and the right graphs as relative miRNA fold change by the 2^-ΔΔCt^ method. Data represents the mean ± SEM. *p<0.05; **p<0.01.

The relative amount of the microRNAs was determined by calculating the difference of Ct values between the Exo and Sn or CFU. In total SLE patient group, we have shown that Exo fraction was significantly increased for all miRNAs compared to Sn fraction, especially miR-146a (6-fold change, p<0.01) (Fig 3). Moreover, when we compare the urine fractions according to the presence of lupus nephritis, as shown in Fig 4A (left), the average of Ct in the control group for the ubiquitously miRNAs were slightly lower in the Exo fraction, whereas specific miRNA were slightly lower in CFU. The mean in the Exo is at least 3.3-fold change (minus 1.8 cycles) higher than in the Sn for ubiquitously miRNAs tested, and the mean in the CFU for miR-200c and miR146a were also at least 3.7 fold-change higher than Sn (Fig 4A, right). CFU fraction was also higher compared to Sn fraction (p<0.05). In addition, we have observed in active LN the highest increase for all miRNAs in urinary Exo fraction (13-fold change for miR-335*, 12-fold change miR-302d and 25-fold change miR-200c, p<0.05; and 57-fold change for miR146a, p<0.01) compared to the values for Sn. That notwithstanding, the highest levels of miR146a were found in CFU (64-fold change, p<0.01) (Fig 4B). In inactive LN, only miR-302d in the Exo fraction was significantly increased in comparison to CFU and Sn (20- and 25-fold change, respectively, p<0.05) (Fig 4C). SLE samples in absence of LN have similar miRNA values among urine fractions (Fig 4D). Together, these data show that the miRNAs tested are contained primarily in exosomes and in low levels outside them. This pattern is especially relevant in the presence of LN.

### Increased miRNAs in the exosome fraction of lupus nephritis patients

**Fig 5:**
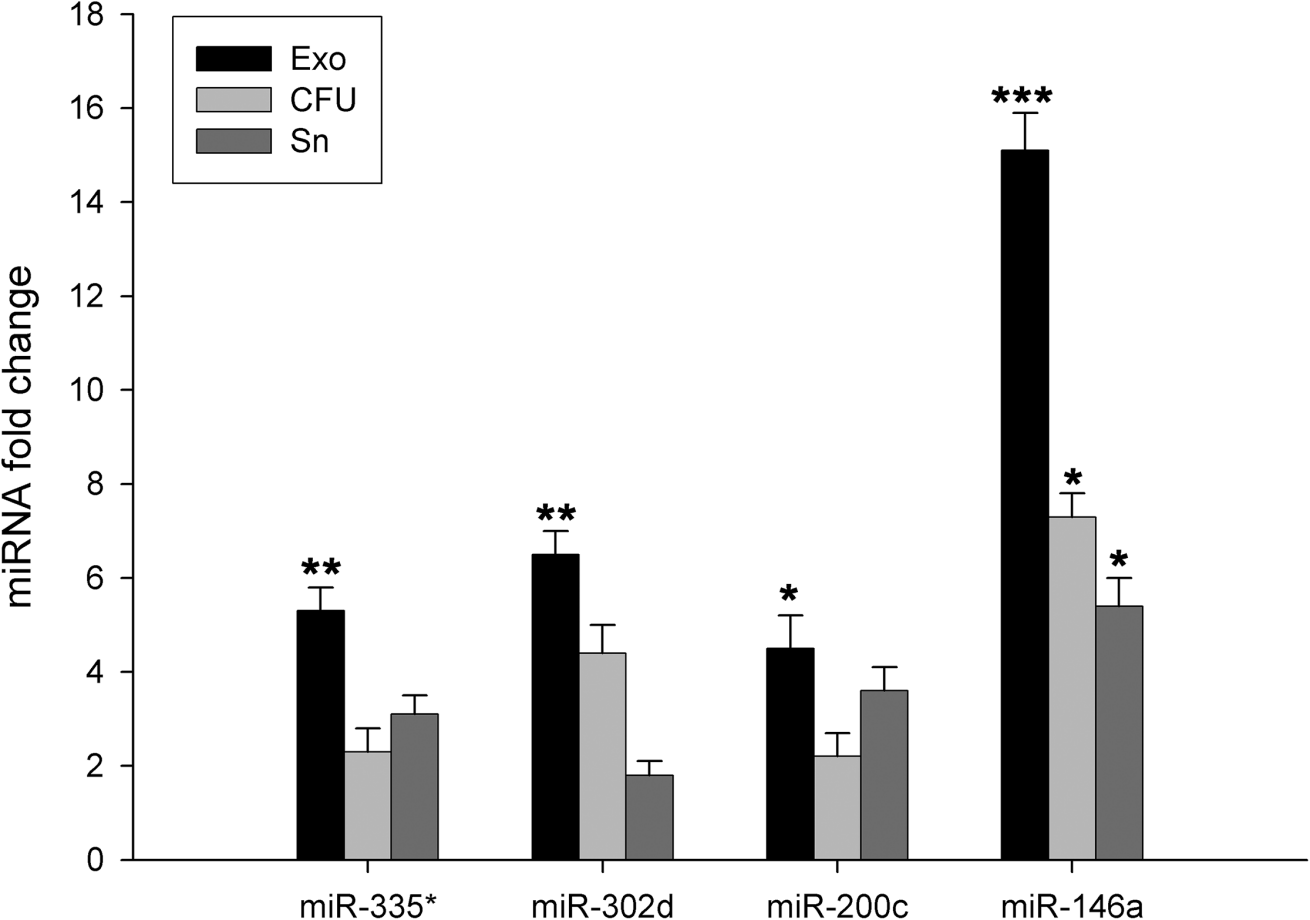
Relative microRNAs expression in urinary fractions from patients with systemic lupus erythematosus respect to controls. Level of the four miRNAs species miR-335*, miR-302d, miR-200c and miR-146a among controls and systemic lupus erythematosus patients in each urinary individual component: (A), exosomes (Exo); (B), cell-free urine (CFU); (C), exosome-depleted supernatant (Sn). The graphs represents relative miRNA fold change by the 2^-ΔΔCt^ method. Data represents the mean ± SEM. *p<0.05; **p<0.01, ***p<0.001.

**Fig 6:**
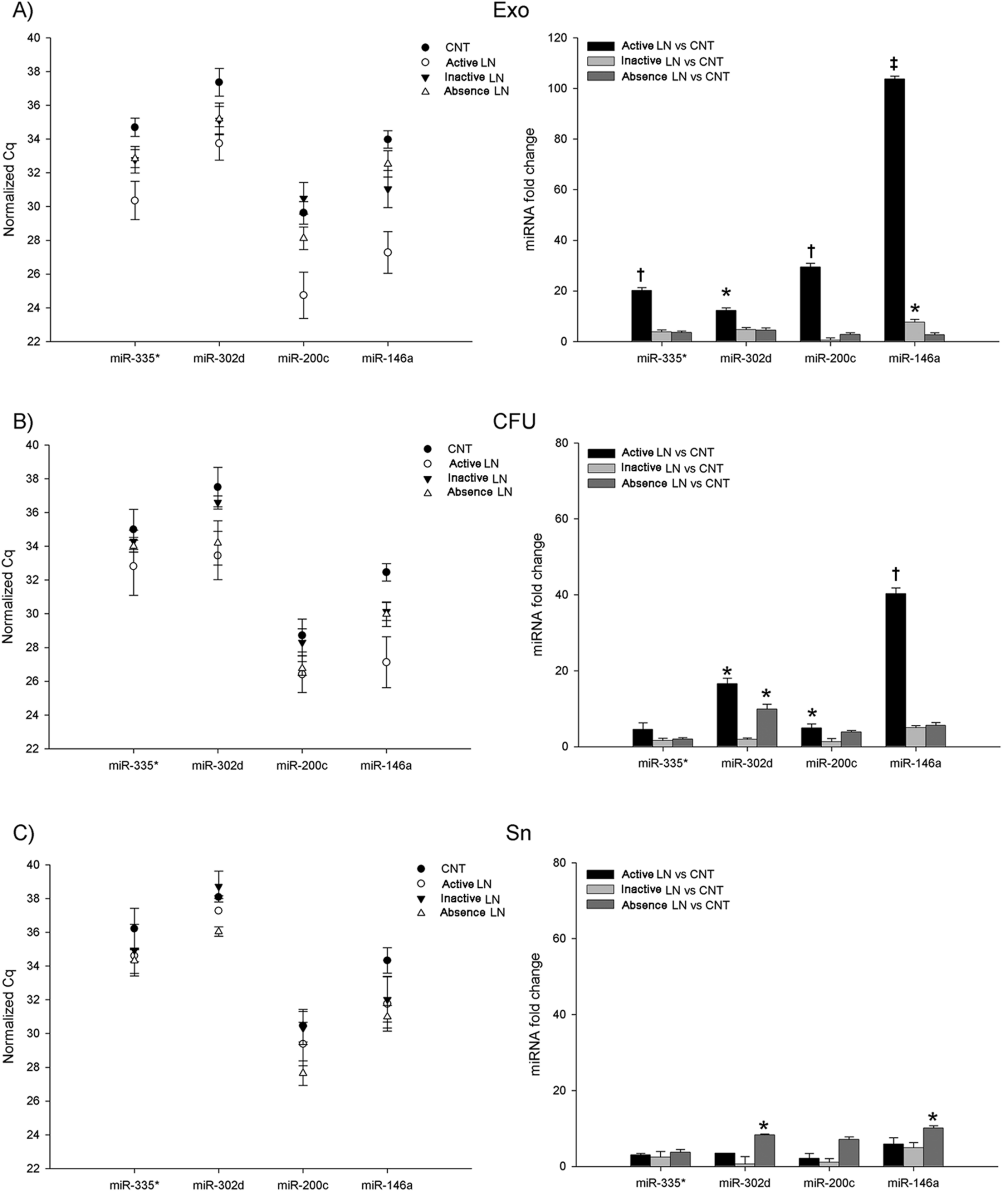
Comparison of miRNAs between control and pathological groups in each individual fractions of urine. The level of the four miRNAs species miR-335*, miR-302d, miR-200c and miR-146a were compared among controls, active lupus nephritis (active LN), inactive lupus nephritis (inactive LN) and systemic lupus erythematosus in the absence of lupus nephritis (absence LN) in each urinary individual component: (A), exosomes (Exo); (B), cell-free urine (CFU); (C), exosome-depleted supernatant (Sn). Left data graphs are expressed as absolute differences (Ct) and right graphs as relative miRNA fold change (2^-ΔΔCt^). Spiked-in cel-miR-39 was used as normalization control for all samples. Each data represents mean ± SEM. *p<0.05; **p<0.01, ***p<0.001 compared to controls.

SLE patient group showed a significant increase for all miRNAs in exosome fraction compared to that in controls, especially for miR-146a (15-fold change, p<0.01). Furthermore, only CFU and Sn fractions of miR-146a were also significantly increased (7-fold change and 5-fold change, p<0.05, respectively) in SLE group (Fig 5). The selected miRNAs were also compared among pathological groups (active LN, inactive LN and SLE in absence of LN) with controls in each urinary fraction (Fig 6). In particular, in the Exo fraction, active LN showed a 20-fold change for miR-335* (p<0.05), a 12-fold change for miR-302d (p<0.05), a 33-fold change for miR-200c (p<0.01) and a 103-fold change for miR-146a (p<0.001) compared to that for controls. Only a significant increase for exosomal miR-146a of 8-fold change, (p<0.05) was found in inactive LN (Fig 6B). Additionally, changes for CFU were from 5-fold (p<0.05) for miR-200c, to 40-fold (p<0.01) for miR-146a in active LN group than in controls. In addition, SLE in absence of LN also showed an increase for miR-302d (10-fold change, p<0.05). Finally, in Sn fraction, only samples from SLE in the absence of LN, had a significant increase for miR-146a and miR-302d (p<0.05) (Fig 6C) compared to controls. These results indicate that in active lupus nephritis the quantity of urinary miRNAs was increased, especially in isolated exosomes, less in CFU and without significant variation in Sn.

### Classification accuracy of the urinary exosomal miRNAs for lupus nephritis

ROC curve analyses were conducted to determine the diagnostic usefulness of the exosomal selected miRNAs for SLE and lupus nephritis. For all the SLE patients (active, inactive and absence LN) and control groups, the AUCs ranged from 0.705 to 0.839. Furthermore, for LN samples (active and inactive), the AUCs ranged from 0.788 to 0.948 (Table 2), with the most discriminatory being the exosomal miRNA-146a (p<0.001). Likewise, only urinary exosomal miRNA-146a had a significant diagnostic role of active LN compared to that for SLE in the absence of LN (AUC 0.960, p<0.01) with a cutoff value of 15.3-fold change (sensitivity 100% and specificity 90%), and between active LN and inactive LN (AUC 0.867, p<0.05) with an optimal cutoff value of 47.2-fold change, 80% sensitivity and 89% specificity.

**Table 2:** ROC curves and the corresponding AUCs of the selected miRNAs in exosomal fraction for all SLE patients (active, inactive and absence of lupus nephritis) or patients with lupus nephritis (active an inactive) compared to control group.

miRNAs	All SLE Patients *vs* Controls					LN *vs* Controls				
	Area	p	Cutoff value (Fold change)	ss (%)	sp (%)	Area	p	Cutoff value (Fold change)	ss (%)	sp (%)
miR-335*	0.822	0.002	1.43	83	75	0.840	0.02	6.48	80	73
**miR-302d**	0.839	0.009	1.67	86	75	0.788	0.06	4.37	80	71
**miR-200c**	0.705	0.04	1.97	69	63	0.813	0.02	2.74	83	63
**miR-146a**	0.764	0.01	2.14	71	61	0.948	0.001	11.25	100	83

LN: lupus nephritis, SLE: systemic lupus erythematosus, sp: specificity, ss: sensitivity.

## Discussion

In the present study, two questions were addressed, if urinary miRNAs are mainly in exosomes and if lupus nephritis (LN) changes the miRNA distribution pattern in urine. As a tool to measure mature miRNAs, individual TaqMan miRNA assays for four common miRNAs, two ubiquitously expressed in human urine (miR302d and miR-335*) [7], and other two described as urine and glomerular tissue biomarkers of renal injury (miR-200c and miR-146a, respectively), were quantified [31,32]. These miRNAs were all readily detectable in the different urine fractions in both patients and healthy controls. Quantification of miRNAs demonstrated that intact miRNAs are enriched in exosome-containing pellet compared to CFU and Sn of urine, confirming that miRNAs were concentrated inside exosomes, although miR146a was slightly higher in CFU rather exosome pellet. This fact could be explain because this urinary fraction contain extracellular vesicles: large vesicles (apoptotic bodies and microvesicles) and small vesicles (exosomes) that transport specific sets of miRNAs. In particular, previous authors have reported that miR-146a is present in apoptotic bodies [33,34], and apoptosis is a pathological process existing in lupus nephritis. Moreover, exosomal miR-302d fraction was more increase than the others miRNAs tested in inactive LN. The miR-302 family has been recently reported that reduce the effects of TGF-β in terms of mesangial expansion and fibrogenesis [35]. Therefore, in our patients with LN, the miR-302d levels could be augmented into urinary exosomes to try to control the pathological effects of TGF-β, especially in inactive LN where normalized renal function is achieved. In summary, the presence of active LN increases the quantity of urinary miRNAs, especially in isolated exosomes, less in CFU and without significant variation in urine Sn. These results would be in concordance with a recent publication in other body fluids that found that the majority of miRNAs detectable in human serum and saliva is concentrated in exosomes [36].

Several studies analysed miRNAs in urine to identify potential biomarkers for renal diseases. Most authors use the urine cellular sediment for miRNA analysis [21,37]. Nevertheless, we have detected a proportion of degraded RNA in urinary cell pellets, perhaps due to the high presence of ribonucleases in the kidney, bladder and urinary tract [38]. Other authors analysed miRNAs or exosomal miRNAs of healthy subjects or of patients with kidney disease [9,26,39]. However, there is no study that has analysed the contribution of exosomal miRNAs to whole urine miRNAs. The present study has determined that miRNAs are found primarily in exosomes in systemic lupus erythematosus, furthermore, in the presence of active LN the fold change of miRNAs in exosomes is markedly superior with respect to the other urine fractions, showing that exosome isolation improves the sensitivity of miRNA quantification from urine specimens. The abundance of miRNAs in urinary exosomes could support the function of the protective membrane enclosure of exosomes that allows the shuttling of genetic material in biological fluids which would otherwise be degraded by RNase activity. Furthermore, urinary exosomes now offer a novel means to obtain information at the nucleic acid level without the need for invasive and expensive biopsy procedures, potentially taking renal biomarker.

Nevertheless, we have found that the highest increase of exosomal miRNAs was in patient group with active LN compared to inactive LN, SLE in absence of LN and controls. Additionally, CFU showed an increase in miRNA levels, but lower than exosome pellet. At variance with non-exosomal urinary RNAs, exosomal miRNAs are in a remarkably stable form. The association between miRNAs and renal disease has been emphasized [24,25,40]. They derive from viable cells from all nephron segments and may provide valuable insight into renal pathophysiology, can display unique expression profiles, distinctive signatures and different exosomal and non-exosomal profiles in human urine in normal and pathological conditions [9,26]. Thus, in LN, a large quantity of miRNAs could be released into urine due to their relevance and implication in a wide range of several pathological mechanisms developed in this renal disease [25,31,41]. Our results showed that the distribution pattern of miRNAs changed in presence of active LN, where levels of miRNAs secreted by exosomes were augmented in lupus compared to controls. In response to various pathophysiological stimulations involved in LN (mesangial expansion, immune complex deposition, inflammation, fibrosis…) cells can actively package miRNA into exosomes and release them into urine, reflecting the renal histopathological change. But not all miRNAs seem to package in the similar way, miR-146a is the most increased compared to the others in SLE and especially in lupus nephritis. This fact would indicate that the increase of miRNA quantity inside exosomes is selective [42], perhaps, due to their role in the cell-to-cell communication, conferring new functional properties to the recipient cell after the acquisition of the exosome genetic material.

Furthermore, the high increase of exosomal miRNAs could be interesting in the diagnosis of SLE because of the important differences found between lupus patients and the control group, and between the different pathological conditions (active or inactive) of lupus patients. ROC curve analyses also revealed a strong relationship between these miRNAs and SLE, mainly in active lupus nephritis, with miRNA-146a being the most discriminatory. In addition, such information will not only increase the number of novel biomarkers for molecular diagnostics and assessment in SLE, but also provide mechanistic insight regarding the pathogenesis and progression of this disease. The miR-146a is involved in renal inflammation, fibrosis, and negatively regulate the interferon-γ pathway [18,43]. Tissue high levels of miR-146a have been reported in glomerulus of patients with LN [32]. All of these findings support a possible role for this miRNA in the pathogenesis of LN.

Several limitations of the present study need to be acknowledged. First, studies involving a high number of miRNAs analysed, a larger patient cohort and patients with more active SLE are needed for further confirmation of urinary exosomal miRNAs as SLE diagnostic tool. Secondly, the inclusion of a different disease control group than SLE, with similar degree of renal impairment, would be interesting to analyse the specificity of these miRNAs for lupus nephritis. Thirdly, functional experimental studies are required to verify and establish the causal association between increased exosomal miRNAs and SLE.

## Conclusion

The current investigation has shown that mature miRNAs are enriched in urinary exosome fractions than in the other components of urine. In the presence of active LN, the quantity of miRNAs increased in cell-free urine and more highly in exosome pellet than that for controls or SLE patients without renal disease. In addition, these results indicated that levels of urinary exosomal miRNAs might be used as new markers of SLE disease. Therefore, these findings, together with the accurate detection of miRNAs by RT-qPCR and the use of urine, a non-invasive method, underscores the attractiveness of exosomal miRNAs in urine as potential biomarkers for renal diseases and could be providing a new therapeutic approach.
